# Dipotassium tris­odium triphosphate, K_2_Na_3_P_3_O_10_

**DOI:** 10.1107/S1600536812014894

**Published:** 2012-04-18

**Authors:** Meryem Moutataouia, Mohammed Lamire, Mohamed Saadi, Lahcen El Ammari

**Affiliations:** a Laboratoire de Physico-Chimie des Matériaux Inorganiques, Faculté des Sciences Aïn Chock, Casablanca, Morocco; b Laboratoire de Chimie du Solide Appliquée, Faculté des Sciences, Université Mohammed V-Agdal, Avenue Ibn Batouta, BP 1014, Rabat, Morocco

## Abstract

The structure of the title compound, K_2_Na_3_P_3_O_10_, is characterized by open chains of three PO_4_ tetra­hedra linked by single oxygen bridges. The P_3_O_10_ groups have crystallographic twofold symmetry, with the central P atom being located on the twofold rotation axis. One of the sodium ions lies on a centre of inversion, whereas all the remaining atoms are in general positions. The structure is isotypic with that of the high-temperature form of Na_5_P_3_O_10_ phase I.

## Related literature

For compounds with related structures, see: Cruickshank (1964 ▶); Davies & Corbridge (1958 ▶); Dyroff (1965 ▶), Wiench *et al.* (1982 ▶); Dymon & King (1951 ▶); Corbridge (1960 ▶).

## Experimental

### 

#### Crystal data

K_2_Na_3_P_3_O_10_
*M* *_r_* = 400.08
Monoclinic, 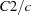

*a* = 9.8866 (4) Å
*b* = 5.6332 (2) Å
*c* = 18.6577 (8) Å
β = 96.199 (3)°
*V* = 1033.03 (7) Å^3^
*Z* = 4
Mo *K*α radiation
μ = 1.55 mm^−1^
*T* = 296 K
0.19 × 0.14 × 0.10 mm

#### Data collection

Bruker APEXII CCD detector diffractometer
Absorption correction: multi-scan (*SADABS*; Sheldrick, 1999 ▶) *T*_min_ = 0.511, *T*_max_ = 0.638
12463 measured reflections
2830 independent reflections
2136 reflections with *I* > 2σ(*I*)
*R*_int_ = 0.032

#### Refinement

*R*[*F* ^2^ > 2σ(*F* ^2^)] = 0.032
*wR*(*F* ^2^) = 0.080
*S* = 1.08
2830 reflections
85 parameters
Δρ_max_ = 0.63 e Å^−3^
Δρ_min_ = −0.97 e Å^−3^

### 

Data collection: *APEX2* (Bruker, 2005 ▶); cell refinement: *SAINT* (Bruker, 2005 ▶); data reduction: *SAINT*; program(s) used to solve structure: *SHELXS97* (Sheldrick, 2008 ▶); program(s) used to refine structure: *SHELXL97* (Sheldrick, 2008 ▶); molecular graphics: *ORTEP-3 for Windows* (Farrugia,1997 ▶) and *DIAMOND* (Brandenburg, 2006 ▶); software used to prepare material for publication: *WinGX* (Farrugia, 1999 ▶).

## Supplementary Material

Crystal structure: contains datablock(s) I, global. DOI: 10.1107/S1600536812014894/bt5872sup1.cif

Structure factors: contains datablock(s) I. DOI: 10.1107/S1600536812014894/bt5872Isup2.hkl

Additional supplementary materials:  crystallographic information; 3D view; checkCIF report

## Figures and Tables

**Fig. 1.**
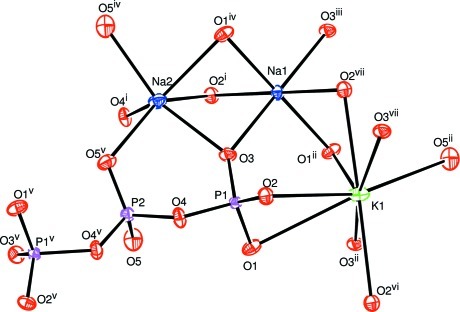
Plot of K2Na3P3O10 crystal structure showing polyhedra linkage. Displacement ellipsoids are drawn at the 50% probability level. Symmetry codes:(i) -x + 1/2, -y + 1/2, -z + 1; (ii) -x, -y, -z + 1; (iii) -x, -y + 1, -z + 1; (iv) x, y + 1, z; (v) -x, y, -z + 1/2; (vi) x - 1/2, y + 1/2, z; (vii) -x + 1/2, y + 1/2, -z + 1/2; (viii) x + 1/2, y - 1/2, z; (ix) x, y - 1, z; (x) -x + 1/2, -y - 1/2, -z + 1.

**Fig. 2.**
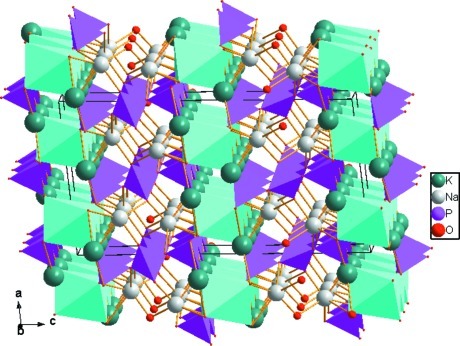
Projection view of the K2Na3P3O10 framework structure showing tunnel running along b direction where the Na1 atoms are located.

## supplementary crystallographic information

### Comment

The present triphosphate was obtained by chance, during the preparation of a mixed pyrophosphate. A bibliographic study of alkali triphosphates like *M*_5_P_3_0_10_ shows that there are few known structures. Thus, in the case of sodium triphosphate, the crystal structures of two anhydrous forms noted Phase I and II were determined by Cruickshank (1964) and Davies & Corbridge (1958) and that of the hexahydrate was performed by Dyroff (1965) and re-examined by Wiench *et al.* (1982).

The structure of dipotassium trisodium triphosphate consists of open chains of three PO_4_ tetrahedra linked by single oxygen bridges. The values of P–P (2.883 Å) distances and P—O—P (125.25°) angles are within the limits generally observed in condensed phosphate crystal chemistry. The internal symmetry of the P_3_0_10_ groups has a twofold symmetry, with the central phosphorus P2 atom being located on a binary axis. Moreover, the Na2 sodium ion lies on the symmetry center whereas all the remaining atoms are in general positions of the *C2/c* space group. The Na2 sodium atom located at Wyckoff position *4c* (1/4, 3/4, 1/2) could be surrounded by a roughly octahedral arrangement of six oxygen atoms and the other sodium and potassium (Na1, K1) atoms are coordinated to six and eight oxygen atoms respectively. The Na2O_6_ octahedra, Na1O_6_ and K1O_8_ polyhedra are connected through the apices to triphosphate groups and form a three-dimensional host lattice (Fig.1). The resulting 3-D framework presents intersecting tunnels running along the [010] and [110] directions, where the six-coordinated Na1^+^ cations are located (Fig.2). The structure of this compound is isotype to that of the high-temperature form of Na_5_P_3_0_10_ phase I (Dymon and King, 1951 and Corbridge, 1960).

### Experimental

The present triphosphate is obtained by chance, during the preparation of a mixture of pyrophosphate. Indeed, the powder phase NaKNiP_2_O_7_ synthesized by wet process is introduced into a platinum crucible, and then gradually heated to a temperature above its melting point (1173 K) for 2 h, followed by slow cooling of the order of 6 K per hour up to 673 K. Then the furnace is shuts down and the cooling is continued until room temperature. Small colourless single crystals of K_2_Na_3_P_3_O_10_ were isolated from the mixtures of phases.

### Refinement

The highest peak and the deepest hole in the final Fourier map are at 0.63 Å and 0.58 Å, respectively, from K1.

### Crystal data

**Table:**

K_2_Na_3_P_3_O_10_	*F*(000) = 784
*M**_r_* = 400.08	*D*_x_ = 2.572 Mg m^−^^3^
Monoclinic, *C*2/*c*	Mo *K*α radiation, λ = 0.71073 Å
Hall symbol: -c 2yc	Cell parameters from 2830 reflections
*a* = 9.8866 (4) Å	θ = 4.2–38.3°
*b* = 5.6332 (2) Å	µ = 1.55 mm^−^^1^
*c* = 18.6577 (8) Å	*T* = 296 K
β = 96.199 (3)°	Block, colourless
*V* = 1033.03 (7) Å^3^	0.19 × 0.14 × 0.10 mm
*Z* = 4	

### Data collection

**Table:**

Bruker APEXII CCD detector diffractometer	2830 independent reflections
Radiation source: fine-focus sealed tube	2136 reflections with *I* > 2σ(*I*)
Graphite monochromator	*R*_int_ = 0.032
ω and φ scans	θ_max_ = 38.3°, θ_min_ = 4.2°
Absorption correction: multi-scan (*SADABS*; Sheldrick, 1999)	*h* = −17→17
*T*_min_ = 0.511, *T*_max_ = 0.638	*k* = −8→9
12463 measured reflections	*l* = −32→32

### Refinement

**Table:**

Refinement on *F*^2^	Primary atom site location: structure-invariant direct methods
Least-squares matrix: full	Secondary atom site location: difference Fourier map
*R*[*F*^2^ > 2σ(*F*^2^)] = 0.032	*w* = 1/[σ^2^(*F*_o_^2^) + (0.0334*P*)^2^ + 0.7541*P*] where *P* = (*F*_o_^2^ + 2*F*_c_^2^)/3
*wR*(*F*^2^) = 0.080	(Δ/σ)_max_ = 0.001
*S* = 1.08	Δρ_max_ = 0.63 e Å^−^^3^
2830 reflections	Δρ_min_ = −0.97 e Å^−^^3^
85 parameters	Extinction correction: *SHELXL97* (Sheldrick, 2008), Fc^*^=kFc[1+0.001xFc^2^λ^3^/sin(2θ)]^-1/4^
0 restraints	Extinction coefficient: 0.0011 (3)

### Special details

**Table:**

Geometry. All s.u.'s (except the s.u. in the dihedral angle between two l.s. planes) are estimated using the full covariance matrix. The cell s.u.'s are taken into account individually in the estimation of s.u.'s in distances, angles and torsion angles; correlations between s.u.'s in cell parameters are only used when they are defined by crystal symmetry. An approximate (isotropic) treatment of cell s.u.'s is used for estimating s.u.'s involving l.s. planes.
Refinement. Refinement of *F*^2^ against all reflections. The weighted *R*-factor *wR* and goodness of fit *S* are based on *F*^2^, conventional *R*-factors *R* are based on *F*, with *F* set to zero for negative *F*^2^. The threshold expression of *F*^2^ > 2σ(*F*^2^) is used only for calculating *R*-factors(gt) *etc*. and is not relevant to the choice of reflections for refinement. *R*-factors based on *F*^2^ are statistically about twice as large as those based on *F*, and *R*- factors based on all data will be even larger.

### Fractional atomic coordinates and isotropic or equivalent isotropic displacement parameters (Å^2^)

**Table:**

	*x*	*y*	*z*	*U*_iso_*/*U*_eq_	
K1	0.09445 (3)	0.27218 (6)	0.57736 (2)	0.02014 (8)	
P1	0.06781 (3)	0.22944 (6)	0.398705 (18)	0.00976 (7)	
P2	0.0000	0.09573 (9)	0.2500	0.01288 (10)	
Na1	0.21820 (7)	−0.30829 (11)	0.32759 (3)	0.01917 (13)	
Na2	0.2500	−0.2500	0.5000	0.01344 (15)	
O1	−0.07276 (11)	0.22433 (19)	0.42318 (6)	0.0184 (2)	
O2	0.15106 (10)	0.44080 (18)	0.42676 (5)	0.01569 (19)	
O3	0.14234 (10)	−0.00428 (18)	0.40638 (6)	0.01571 (19)	
O4	0.04508 (11)	0.28577 (18)	0.31142 (5)	0.01663 (19)	
O5	−0.12023 (12)	−0.0419 (2)	0.26927 (6)	0.0231 (2)	

### Atomic displacement parameters (Å^2^)

**Table:**

	*U*^11^	*U*^22^	*U*^33^	*U*^12^	*U*^13^	*U*^23^
K1	0.01413 (13)	0.01460 (13)	0.03200 (18)	0.00043 (10)	0.00389 (11)	0.00128 (11)
P1	0.00897 (13)	0.00981 (14)	0.01046 (14)	0.00010 (10)	0.00091 (10)	−0.00060 (10)
P2	0.0142 (2)	0.0135 (2)	0.0108 (2)	0.000	0.00129 (15)	0.000
Na1	0.0199 (3)	0.0173 (3)	0.0212 (3)	0.0060 (2)	0.0062 (2)	0.0047 (2)
Na2	0.0132 (3)	0.0131 (4)	0.0139 (3)	−0.0003 (3)	0.0008 (3)	−0.0006 (3)
O1	0.0136 (4)	0.0187 (5)	0.0244 (5)	−0.0006 (4)	0.0090 (4)	−0.0020 (4)
O2	0.0151 (4)	0.0140 (4)	0.0174 (4)	−0.0028 (3)	−0.0007 (3)	−0.0041 (3)
O3	0.0162 (4)	0.0126 (4)	0.0180 (4)	0.0044 (3)	0.0003 (3)	0.0015 (3)
O4	0.0239 (5)	0.0159 (4)	0.0095 (4)	−0.0021 (4)	−0.0010 (3)	0.0012 (3)
O5	0.0238 (5)	0.0246 (5)	0.0216 (5)	−0.0107 (4)	0.0056 (4)	0.0005 (4)

### Geometric parameters (Å, º)

**Table:**

K1—O2^i^	2.7960 (11)	P2—O4	1.5961 (11)
K1—O1^ii^	2.8051 (11)	P2—O4^iv^	1.5961 (11)
K1—O3^ii^	2.8291 (11)	Na1—O5^v^	2.4172 (14)
K1—O1^iii^	2.8443 (11)	Na1—O3	2.4278 (12)
K1—O3^i^	2.8987 (11)	Na1—O5^iv^	2.4655 (13)
K1—O2^iii^	2.9106 (11)	Na1—O2^vi^	2.4753 (12)
K1—O2	3.0740 (11)	Na1—O1^v^	2.5864 (13)
K1—O5^ii^	3.1272 (12)	Na1—O4^vi^	2.8527 (13)
P1—O3	1.5080 (10)	Na2—O2^vi^	2.3599 (10)
P1—O2	1.5086 (10)	Na2—O2^i^	2.3599 (10)
P1—O1	1.5092 (11)	Na2—O1^ii^	2.3850 (10)
P1—O4	1.6506 (10)	Na2—O1^v^	2.3850 (10)
P2—O5	1.4951 (11)	Na2—O3	2.3871 (10)
P2—O5^iv^	1.4951 (11)	Na2—O3^vii^	2.3871 (10)
			
O2^i^—K1—O1^ii^	68.95 (3)	O3—P1—O4	105.94 (6)
O2^i^—K1—O3^ii^	122.11 (3)	O2—P1—O4	101.64 (6)
O1^ii^—K1—O3^ii^	53.46 (3)	O1—P1—O4	105.69 (6)
O2^i^—K1—O1^iii^	119.70 (3)	O5—P2—O5^iv^	117.54 (10)
O1^ii^—K1—O1^iii^	171.32 (4)	O5—P2—O4	110.04 (6)
O3^ii^—K1—O1^iii^	117.99 (3)	O5^iv^—P2—O4	110.66 (6)
O2^i^—K1—O3^i^	52.85 (3)	O5—P2—O4^iv^	110.66 (6)
O1^ii^—K1—O3^i^	121.17 (3)	O5^iv^—P2—O4^iv^	110.04 (6)
O3^ii^—K1—O3^i^	166.73 (4)	O4—P2—O4^iv^	95.75 (8)
O1^iii^—K1—O3^i^	67.49 (3)	O5^v^—Na1—O3	156.77 (5)
O2^i^—K1—O2^iii^	171.11 (4)	O5^v^—Na1—O5^iv^	103.17 (3)
O1^ii^—K1—O2^iii^	119.38 (3)	O3—Na1—O5^iv^	83.79 (4)
O3^ii^—K1—O2^iii^	66.53 (3)	O5^v^—Na1—O2^vi^	105.62 (4)
O1^iii^—K1—O2^iii^	51.95 (3)	O3—Na1—O2^vi^	79.92 (4)
O3^i^—K1—O2^iii^	119.27 (3)	O5^iv^—Na1—O2^vi^	141.23 (5)
O2^i^—K1—O2	81.62 (3)	O5^v^—Na1—O1^v^	80.29 (4)
O1^ii^—K1—O2	109.04 (3)	O3—Na1—O1^v^	78.96 (4)
O3^ii^—K1—O2	119.82 (3)	O5^iv^—Na1—O1^v^	133.13 (5)
O1^iii^—K1—O2	73.13 (3)	O2^vi^—Na1—O1^v^	77.52 (4)
O3^i^—K1—O2	72.92 (3)	O5^v^—Na1—O4^vi^	86.22 (4)
O2^iii^—K1—O2	92.17 (3)	O3—Na1—O4^vi^	114.11 (4)
O2^i^—K1—O5^ii^	82.10 (3)	O5^iv^—Na1—O4^vi^	103.10 (4)
O1^ii^—K1—O5^ii^	65.69 (3)	O2^vi^—Na1—O4^vi^	54.20 (3)
O3^ii^—K1—O5^ii^	70.56 (3)	O1^v^—Na1—O4^vi^	123.75 (4)
O1^iii^—K1—O5^ii^	114.55 (3)	O2^vi^—Na2—O2^i^	180.0
O3^i^—K1—O5^ii^	96.19 (3)	O2^vi^—Na2—O1^ii^	96.16 (4)
O2^iii^—K1—O5^ii^	103.88 (3)	O2^i^—Na2—O1^ii^	83.85 (4)
O2—K1—O5^ii^	163.68 (3)	O2^vi^—Na2—O1^v^	83.85 (4)
O2^i^—K1—O1	108.73 (3)	O2^i^—Na2—O1^v^	96.15 (4)
O1^ii^—K1—O1	83.10 (3)	O1^ii^—Na2—O1^v^	180.000 (1)
O3^ii^—K1—O1	72.27 (3)	O2^vi^—Na2—O3	83.11 (3)
O1^iii^—K1—O1	92.86 (3)	O2^i^—Na2—O3	96.89 (3)
O3^i^—K1—O1	120.50 (3)	O1^ii^—Na2—O3	96.09 (4)
O2^iii^—K1—O1	70.83 (3)	O1^v^—Na2—O3	83.91 (4)
O2—K1—O1	47.59 (3)	O2^vi^—Na2—O3^vii^	96.89 (3)
O5^ii^—K1—O1	141.07 (3)	O2^i^—Na2—O3^vii^	83.11 (3)
O3—P1—O2	114.42 (6)	O1^ii^—Na2—O3^vii^	83.91 (4)
O3—P1—O1	114.27 (6)	O1^v^—Na2—O3^vii^	96.09 (4)
O2—P1—O1	113.31 (6)	O3—Na2—O3^vii^	180.00 (4)

Symmetry codes: (i) −*x*+1/2, −*y*+1/2, −*z*+1; (ii) −*x*, −*y*, −*z*+1; (iii) −*x*, −*y*+1, −*z*+1; (iv) −*x*, *y*, −*z*+1/2; (v) *x*+1/2, *y*−1/2, *z*; (vi) *x*, *y*−1, *z*; (vii) −*x*+1/2, −*y*−1/2, −*z*+1.
